# A genome-reduced *Corynebacterium glutamicum* derivative discloses a hidden pathway relevant for 1,2-propanediol production

**DOI:** 10.1186/s12934-024-02337-w

**Published:** 2024-02-24

**Authors:** Daniel Siebert, Erich Glawischnig, Marie-Theres Wirth, Mieke Vannahme, Álvaro Salazar-Quirós, Annette Weiske, Ezgi Saydam, Dominik Möggenried, Volker F. Wendisch, Bastian Blombach

**Affiliations:** 1 Microbial Biotechnology, Campus Straubing for Biotechnology and Sustainability, Technical University of Munich, Straubing, Germany; 2 SynBiofoundry@TUM, Technical University of Munich, Straubing, Germany; 3 Chair of Genetics of Prokaryotes, Faculty of Biology & CeBiTec, Bielefeld University, Bielefeld, Germany

**Keywords:** *Corynebacterium glutamicum*, Genome reduction, Chassis organism, 1,2-propanediol, Mycothiol, Methylglyoxal, Lactoylmycothiol

## Abstract

**Background:**

1,2-propanediol (1,2-PDO) is widely used in the cosmetic, food, and drug industries with a worldwide consumption of over 1.5 million metric tons per year. Although efforts have been made to engineer microbial hosts such as *Corynebacterium glutamicum* to produce 1,2-PDO from renewable resources, the performance of such strains is still improvable to be competitive with existing petrochemical production routes.

**Results:**

In this study, we enabled 1,2-PDO production in the genome-reduced strain *C. glutamicum* PC2 by introducing previously described modifications. The resulting strain showed reduced product formation but secreted 50 ± 1 mM d-lactate as byproduct. *C. glutamicum* PC2 lacks the d-lactate dehydrogenase which pointed to a yet unknown pathway relevant for 1,2-PDO production. Further analysis indicated that in *C. glutamicum* methylglyoxal, the precursor for 1,2-PDO synthesis, is detoxified with the antioxidant native mycothiol (MSH) by a glyoxalase-like system to lactoylmycothiol and converted to d-lactate which is rerouted into the central carbon metabolism at the level of pyruvate. Metabolomics of cell extracts of the empty vector-carrying wildtype, a 1,2-PDO producer and its derivative with inactive d-lactate dehydrogenase identified major mass peaks characteristic for lactoylmycothiol and its precursors MSH and glucosaminyl-myo-inositol, whereas the respective mass peaks were absent in a production strain with inactivated MSH synthesis. Deletion of *mshA*, encoding MSH synthase, in the 1,2-PDO producing strain *C. glutamicum* Δ*hdpA*Δ*ldh*(pEKEx3-*mgsA*-*yqhD*-*gldA*) improved the product yield by 56% to 0.53 ± 0.01 mM_1,2−PDO_ mM_glucose_^−1^ which is the highest value for *C. glutamicum* reported so far.

**Conclusions:**

Genome reduced-strains are a useful basis to unravel metabolic constraints for strain engineering and disclosed in this study the pathway to detoxify methylglyoxal which represents a precursor for 1,2-PDO production. Subsequent inactivation of the competing pathway significantly improved the 1,2-PDO yield.

**Supplementary Information:**

The online version contains supplementary material available at 10.1186/s12934-024-02337-w.

## Introduction

The C3-diols 1,2- and 1,3-propanediol (PDO) are important building blocks and are widely used in the polymer, food, cosmetic and drug industry [1, 2]. For 1,3-PDO and 1,2-PDO a global market size of around 1.4 billion and 0.4 billion US dollars is expected in the next years [2, 3]. Both diols are mainly produced from fossil fuels, but bio-based production processes, utilizing renewable resources, are favorable to tackle the concerns of climate change and the limited availability of fossil resources. Notably, first commercial microbial processes for 1,3-PDO production are readily available [4]. Although several natural 1,2-PDO producers are known and established microbial systems have been extensively engineered for its production, a sustainable process at the industrial level is still missing [2].

**Fig. 1:**
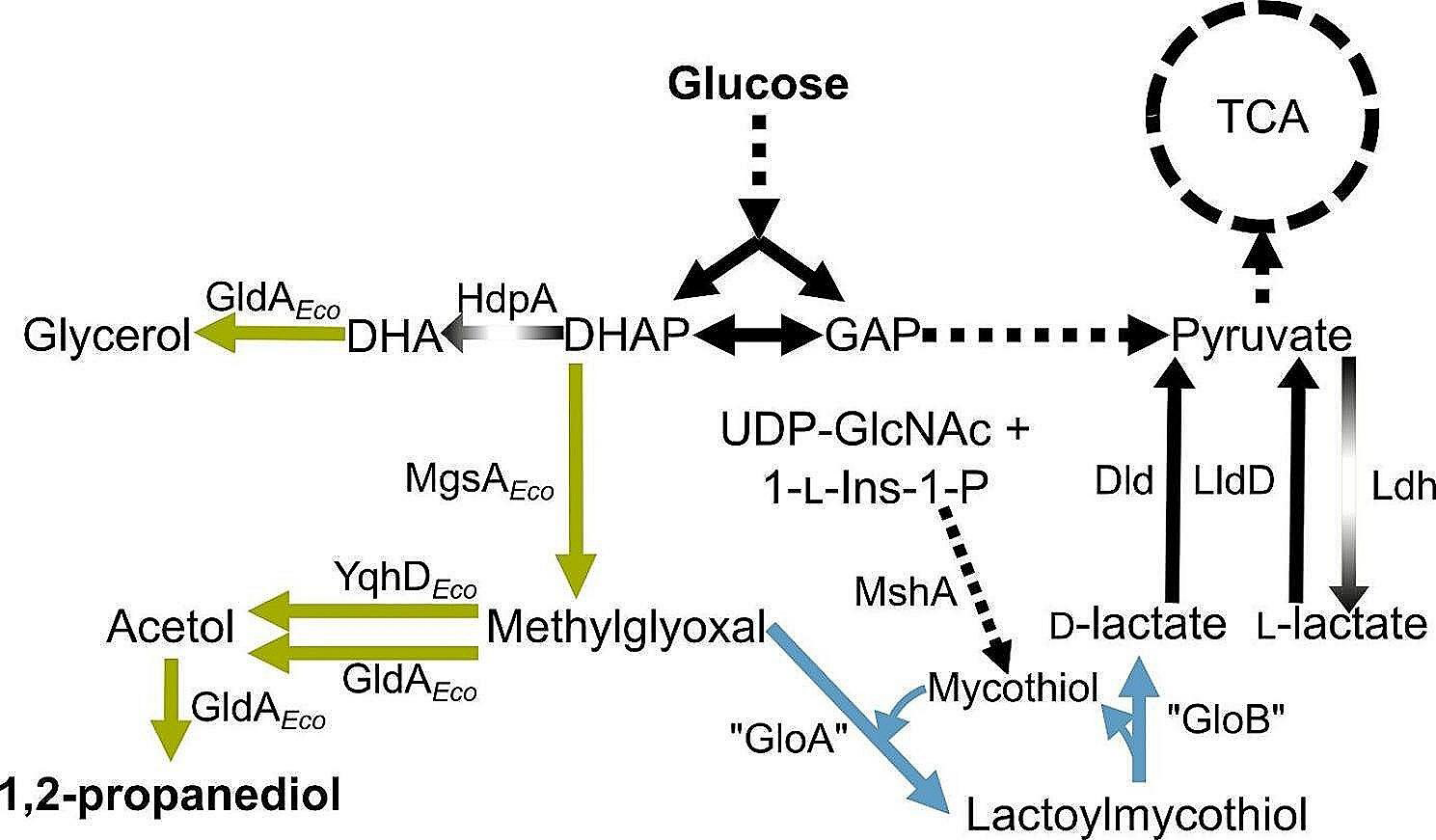
Overview of the 1,2-PDO pathway introduced into *C. glutamicum* [22], including the proposed bypass from methylglyoxal to d-lactate and pyruvate via lactoylmycothiol in blue. Black arrows represent native pathways; dotted arrows indicate more than one reaction; green and disrupted arrows represent heterologously expressed proteins and deletions of gene sequences of mentioned proteins, respectively. Abbreviations: TCA, tricarboxylic acid cycle; DHA, dihydroxyacetone; DHAP, dihydroxyacetone phosphate; GAP, glyceraldehyde 3-phosphate; UDP-GlcNAc, uridine diphosphate-N-acetylglucosamine; 1-l-Ins-1-P, 1-l-*myo*-inositol 1-phosphate; GldA, glycerol dehydrogenase (from *E. coli*); HdpA, dihydroxyacetone phosphate phosphatase; MgsA, methylglyoxal synthase (from *E. coli*); Dld, quinone-dependent d-lactate dehydrogenase; LldD, quinone-dependent l-lactate dehydrogenase; Ldh, NAD-dependent l-lactate dehydrogenase; YqhD, aldehyde reductase (from *E. coli*); MshA, glycosyltransferase; “GloA”, proposed lactoylglutathione lyase homolog in *C. glutamicum*; “GloB”, proposed hydroxyacylglutathione hydrolase homolog in *C. glutamicum*

*Corynebacterium glutamicum* is a facultative anaerobic Gram-positive soil bacterium which is generally recognized as safe (GRAS), robust, and grows with several sugars, organic acids and phenolic compounds as single or combined carbon and energy sources [5–10]. Ample knowledge about the physiology, metabolic and regulatory networks has been gathered and a versatile toolbox for genetic engineering is available [6, 11–14]. This bacterium is known as industrial powerhouse for the production of amino acids such as l-glutamate and l-lysine at a scale of 6 million tons per year [5]. Moreover, sophisticated metabolic engineering approaches extended the product portfolio rapidly [15], also to diols such as 2,3-butanediol [16–19], 1,3-PDO [20] and 1,2-PDO [21]. Production of 1,2-PDO was initially achieved by heterologous expression of the *Escherichia coli* gene *mgsA* (encoding methylglyoxal synthase) and overexpression of a putative aldo-keto reductase (probably functioning as a methylglyoxal reductase) (Fig. 1; [21]). Biosynthesis was further improved by additional heterologous expression of the *E. coli* genes *gldA* (encoding glycerol dehydrogenase) and *yqhD* (encoding aldehyde reductase). Deletions of *hdpA* (encoding dihydroxyacetone phosphate phosphatase) and *ldh* (encoding l-lactate dehydrogenase) avoided the secretion of the side-products lactate and glycerol and improved 1,2-PDO production further (Fig. 1; [22]).

Methylglyoxal is a key intermediate in the 1,2-PDO production route, but also represents a potent cytotoxic compound which reacts with arginine, lysine, and cysteine residues and might lead to protein inactivation [23, 24]. Consequently, methylglyoxal is in many organisms detoxified to d-lactate by a GloAB-mediated glyoxalase system with the tripeptide glutathione (GSH). d-lactate is further oxidized to pyruvate which is subsequently channeled back into the central carbon metabolism (Fig. 1) [23, 24]. A characteristic of *Actinobacteria*, such as *C. glutamicum*, is the absence of GSH biosynthesis. However, in these organisms the low molecular weight thiol and non-enzymatic antioxidant mycothiol (MSH) is synthesized for detoxification instead [25]. MSH is synthezised from the intermediates myo-inositol and *N*-acetyl-glucosamine via glucosaminyl-myo-inositol by five enzymatic steps encoded by the genes *mshA*, *mshA2*, *mshB*, *mshC* and *mshD* [26]. However, no GloAB homologs have been identified in *C. glutamicum* and the mechanism for detoxification of methylglyoxal is elusive so far. Besides MSH as antioxidant, the transcriptional regulator OxyR plays an important role in the oxidative stress response in *C. glutamicum*. Under unstressed conditions this master regulator represses genes encoding enzymes such as catalase. As a result, an *oxyR* deletion mutant shows increased resistance to H_2_O_2_ [27].

The concept of genome reduction to improve physiological characteristics and create optimized hosts for the industrial environment has been applied to many organisms [28] as well as to *C. glutamicum* [29, 30]. In a systematic top-down approach all genes of the *C. glutamicum* genome were ranked by their relevance for growth in minimal medium with glucose as sole carbon and energy source. The proposed gene clusters were individually deleted and the genome-reduced strains (GRS) were screened for their growth phenotype [29]. In a follow-up study the deletions of the most promising genomic regions were combined in a stepwise manner, leading to the pre-chassis strains PC1 and PC2, with 8.5% and 12.6% reduced genomes, respectively. While both of these strains grew like the wildtype, the finally engineered chassis strain C1 (13.4% genome-reduction) showed a growth deficit on acetate due to an unwanted mutation in the promoter of the *ramA* gene, which was repaired yielding the final genome-reduced derivative C1* [30]. Recently, some intermediate strains of the aforementioned genome-reduction approach were utilized to screen for improved heterologous cutinase secretion in *C. glutamicum* [31].

In this study, we introduced known genetic modifications to enable 1,2-PDO production [22] into the genome-reduced strain *C. glutamicum* PC2 [30] and its parental strain GRS [29] (in later studies also called CR099). Only in the PC2 genetic background, we observed the formation of d-lactate as byproduct, which was attributed to the lack of the respective d-lactate dehydrogenase and pointed to a yet unknown pathway relevant for 1,2-PDO production. Further analysis indicated that in *C. glutamicum* methylglyoxal, the precursor for 1,2-PDO synthesis, is converted with MSH by a glyoxalase-like system to lactoylmycothiol and further to d-lactate which is channeled back into the central carbon metabolism on the level of pyruvate (Fig. 1).

## Results

### Reconstruction of the 1,2-PDO production pathway in the *C. glutamicum* strains GRS and PC2

**Fig. 2:**
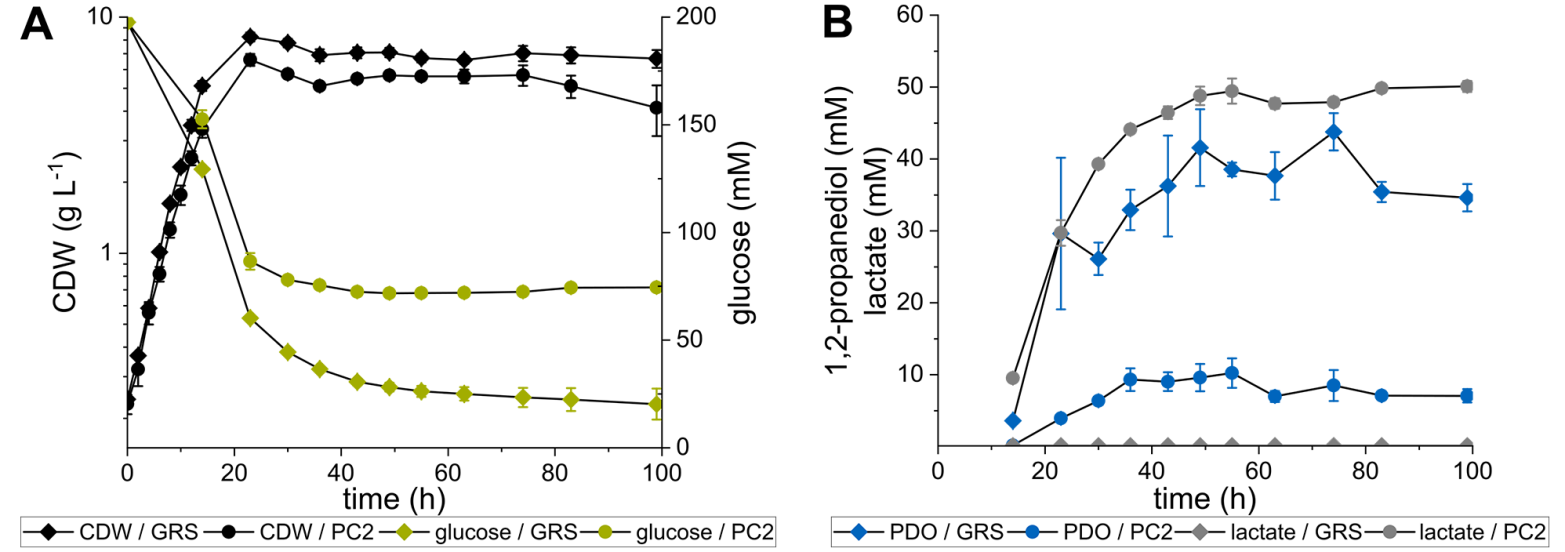
(**A**) Growth (black) and glucose consumption (green) and (**B**) 1,2-PDO (blue) and lactate (grey) accumulation of the strains *C. glutamicum* GRSΔ*hdpA*Δ*ldh*(pEKEx3-*mgsA*-*yqhD*-*gldA*) (GRS) and PC2Δ*hdpA*Δ*ldh*(pEKEx3-*mgsA*-*yqhD*-*gldA*) (PC2) in shaking flasks with modified CGXII minimal medium. Error bars represent the standard deviation of the mean values of three biological replicates

In a former study, it was shown that the deletion of *hdpA* and *ldh* and heterologous expression of the *E. coli* genes *mgsA*, *yqhD* and *gldA* led to the highest production of 1,2-PDO with *C. glutamicum* reported yet [22]. To analyze the production capacity of the genome-reduced strains *C. glutamicum* PC2 [30] and its parental strain GRS [29], the named genetic modifications were introduced into both strains. The resulting strains *C. glutamicum* GRSΔ*hdpA*Δ*ldh* and PC2Δ*hdpA*Δ*ldh*, each carrying the plasmid pEKEx3-*mgsA*-*yqhD*-*gldA* were cultivated in modified CGXII minimal medium with glucose as sole carbon and energy source [22]. Compared to GRS, the strain with PC2 genetic background showed a similar growth rate (0.20 ± 0.01 vs. 0.22 ± 0.01 h^-1^), accumulated less biomass (max. CDW 6.60 ± 0.38 g L^-1^ vs. 8.25 ± 0.36) and exhibited a decreased glucose consumption (178 mM ± 7 vs. 126 ± 1 mM consumed) (Fig. 2A). While *C. glutamicum* GRSΔ*hdpA*Δ*ldh*(pEKEx3-*mgsA*-*yqhD*-*gldA*) produced up to 44 ± 3 mM 1,2-PDO (Y_P/S_ = 0.25 ± 0.01 mol mol^-1^), only 10 ± 2 mM (Y_P/S_ = 0.08 ± 0.02 mol mol^-1^) were secreted by *C. glutamicum* PC2Δ*hdpA*Δ*ldh*(pEKEx3-*mgsA*-*yqhD*-*gldA*) (Fig. 2B). Interestingly, the PC2-based strain produced up to 50 ± 1 mM lactate whereas for the GRS-derivative no lactate was determined in the supernatant (Fig. 2B). With the applied HPLC method it was not possible to distinguish between l- and d-lactate. However, since in both strains the *ldh* gene encoding l-lactate dehydrogenase is deleted, we speculated that d-lactate is secreted by *C. glutamicum* PC2Δ*hdpA*Δ*ldh*(pEKEx3-*mgsA*-*yqhD*-*gldA*). Notably, compared to the GRS strain, the genome-reduced variant PC2 additionally lacks the *dld* gene encoding a quinone-dependent d-lactate dehydrogenase [32], which might be a reasonable explanation for the observed phenotype.

### Impact of *dld* on the production of 1,2-propanediol in *C. glutamicum*

**Fig. 3:**
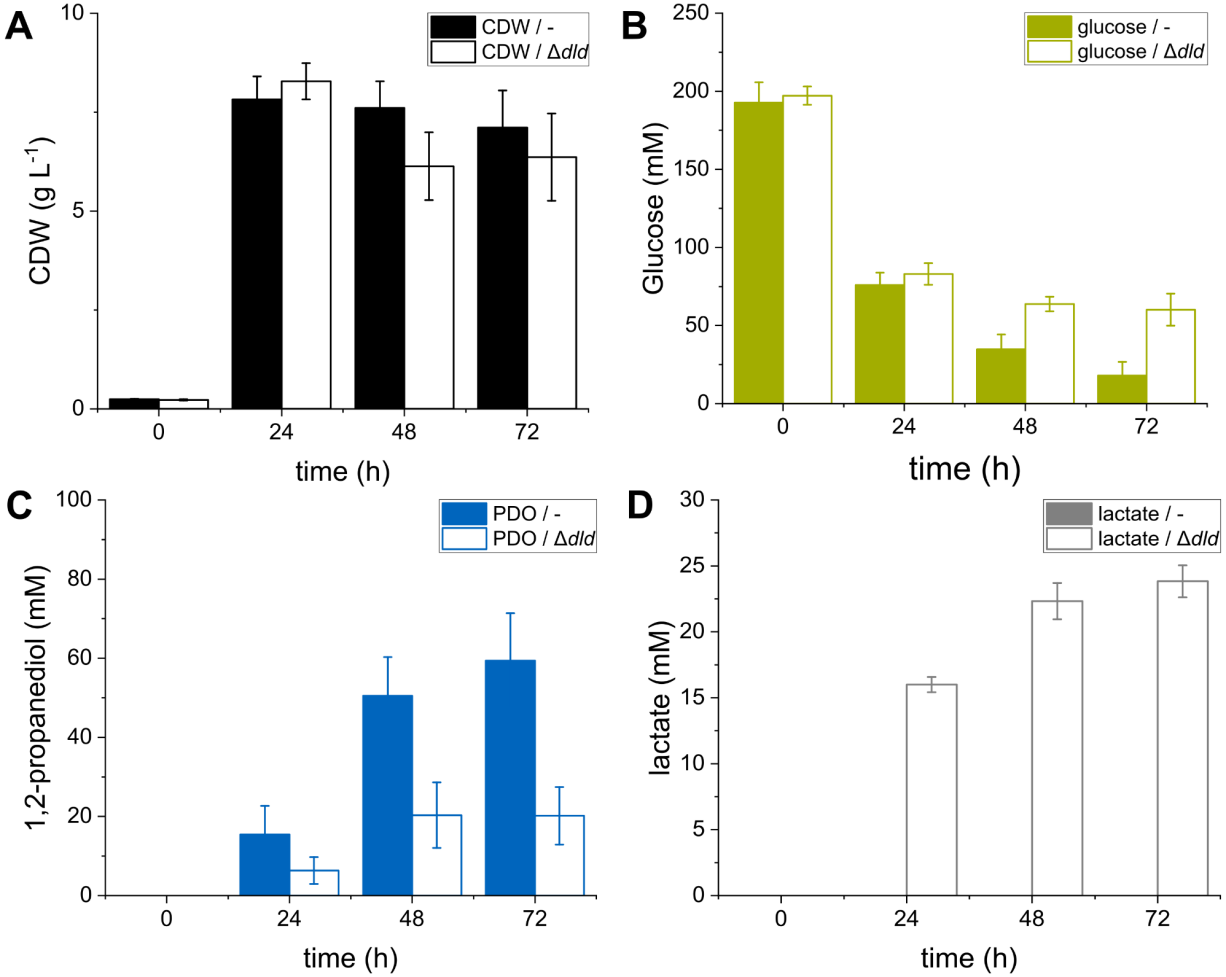
(**A**) Growth (black), (**B**) glucose consumption (green), (**C**) 1,2-PDO (blue) and (**D**) lactate (grey) accumulation of the strains *C. glutamicum* Δ*hdpA*Δ*ldh*(pEKEx3-*mgsA*-*yqhD*-*gldA*) (-) and *C. glutamicum* Δ*hdpA*Δ*ldh*Δ*dld*(pEKEx3-*mgsA*-*yqhD*-*gldA*) (Δ*dld*) in shaking flasks with modified CGXII minimal medium. Error bars represent the standard deviation of the mean values of three biological replicates

To clarify if the *dld* gene has a yet unknown effect on 1,2-PDO production in *C. glutamicum*, we deleted the gene in *C. glutamicum* Δ*hdpA*Δ*ldh*(pEKEx3-*mgsA*-*yqhD*-*gldA*). The resulting strain *C. glutamicum* Δ*hdpA*Δ*ldh*Δ*dld*(pEKEx3-*mgsA*-*yqhD*-*gldA*) and its parental strain were cultivated in shaking flasks with modified CGXII medium and glucose as sole carbon and energy source. The additional deletion of *dld* led to a slightly decreased biomass concentration and reduced glucose consumption (Fig. 3A and B). Further, *C. glutamicum* Δ*hdpA*Δ*ldh*Δ*dld*(pEKEx3-*mgsA*-*yqhD*-*gldA*) secreted up to 24 ± 1 mM lactate and produced three times less 1,2-PDO compared to the parental strain (59 ± 12 mM as compared to 20 ± 7 mM; Fig. 3C and D). The lactate concentrations in the culture supernatants determined via HPLC were confirmed and identified to be d-lactate by a specific enzyme assay (Figure S1). These findings are in agreement with the results obtained with the chassis strain PC2, confirming the relevance of d-lactate dehydrogenase for 1,2-PDO production and hint towards a not yet known pathway in *C. glutamicum* which causes a degradation of the main product or an intermediate of the production pathway.

### Inactivation of mycothiol biosynthesis improves 1,2-PDO production

**Fig. 4:**
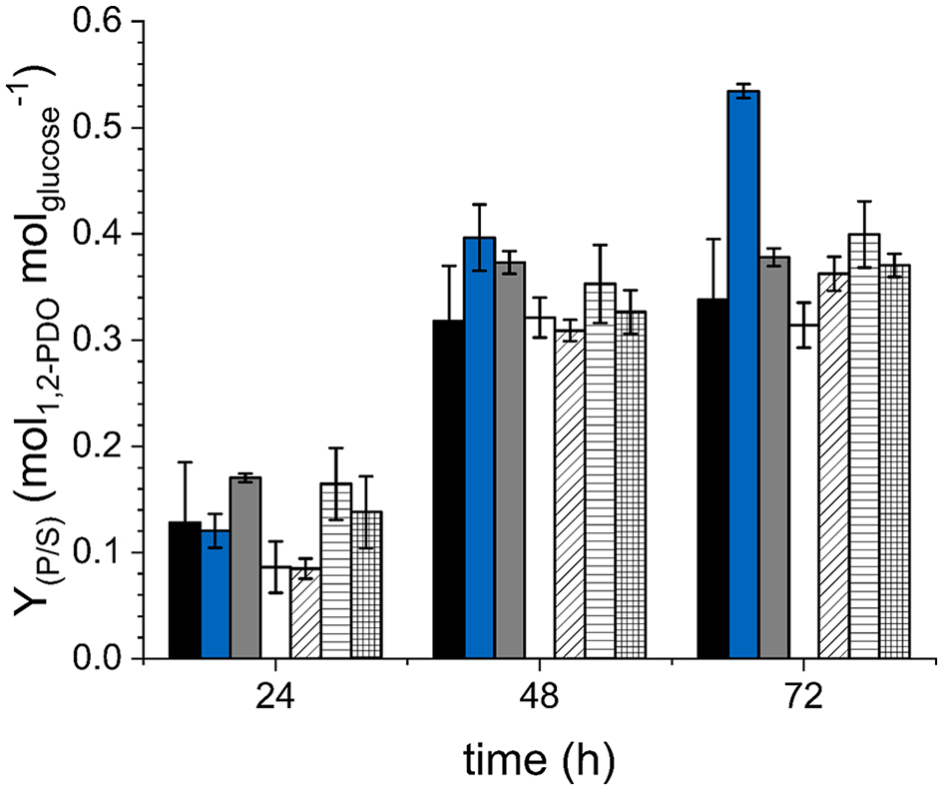
Glucose-specific 1,2-PDO yields of *C. glutamicum* Δ*hdpA*Δ*ldh*(pEKEx3-*mgsA*-*yqhD*-*gldA*) (-), *C. glutamicum* Δ*hdpA*Δ*ldh*Δ*mshA*(pEKEx3-*mgsA*-*yqhD*-*gldA*) (Δ*mshA*), *C. glutamicum* Δ*hdpA*Δ*ldh*Δcg1426(pEKEx3-*mgsA*-*yqhD*-*gldA*) (Δcg1426), *C. glutamicum* Δ*hdpA*Δ*ldh*Δcg1073(pEKEx3-*mgsA*-*yqhD*-*gldA*) (Δcg1073), *C. glutamicum* Δ*hdpA*Δ*ldh*Δcg0071(pEKEx3-*mgsA*-*yqhD*-*gldA*) (Δcg0071), *C. glutamicum* Δ*hdpA*Δ*ldh*Δcg1482(pEKEx3-*mgsA*-*yqhD*-*gldA*) (Δcg1482) and *C. glutamicum* Δ*hdpA*Δ*ldh*Δcg1856(pEKEx3-*mgsA*-*yqhD*-*gldA*) (Δcg1856) cultivated in shaking flasks with modified CGXII minimal medium and glucose as sole carbon and energy source. Error bars represent the standard deviation of the mean values of three biological replicates

We speculated that methylglyoxal might be converted to d-lactate by a MSH-dependent system similar to the glyoxalase system acting with GSH. To investigate this, we identified several targets potentially involved in the detoxification of methylglyoxal in *C. glutamicum*: deletion of the *mshA* gene encoding a glycosyltransferase to avoid MSH biosynthesis [25]; inactivation of the Xi-class mycothiol S-transferase [33] by deletion of the gene cg1426 (NCgl1216/*mstX*); deletion of the putative *gloA* and *gloB* homologs cg1073 as well as cg0071, cg1482 and cg1856, which were identified by annotations in the databases CoryneRegNet 7 [34] and Kyoto Encyclopedia of Genes and Genomes (KEGG) [35]. These deletions were introduced individually into *C. glutamicum* Δ*hdpA*Δ*ldh*, followed by the transformation of the newly constructed strains with the plasmid pEKEx3-*mgsA*-*yqhD*-*gldA*. The resulting strains were cultivated in shaking flasks with modified CGXII minimal medium and glucose as sole carbon and energy source. Neither inactivation of the genes putatively encoding GloAB homologs nor MstX led to a significant change in formed biomass, glucose consumption or product yield (*p* > 0.1) compared to the parental strain *C. glutamicum* Δ*hdpA*Δ*ldh*(pEKEx3-*mgsA*-*yqhD*-*gldA*) which showed a Y_P/S_ of 0.34 ± 0.06 mM_1,2−PDO_ mM_glucose_^−1^ after 72 h of fermentation (Fig. 4 and S2). In contrast, deletion of the *mshA* gene resulted in a 20% reduced biomass formation after 72 h and impaired glucose consumption, however, improved the Y_P/S_ significantly (*p* < 0.01) (Fig. 4 and S2). *C. glutamicum* Δ*hdpA*Δ*ldh*Δ*mshA*(pEKEx3-*mgsA*-*yqhD*-*gldA*) showed a Y_P/S_ of 0.53 ± 0.01 mM_1,2−PDO_ mM_glucose_^−1^ which is 56% higher compared to *C. glutamicum* Δ*hdpA*Δ*ldh*(pEKEx3-*mgsA*-*yqhD*-*gldA*) (Fig. 4), while no lactate was formed. Because the *mshA* deletion abolishes MSH biosynthesis (see below), the improved Y_P/S_ of *C. glutamicum* Δ*hdpA*Δ*ldh*Δ*mshA*(pEKEx3-*mgsA*-*yqhD*-*gldA*) might be a result of increased metylglyoxal availability and/or also of an overall elevated oxidative stress level. To investigate this effect, we deleted and overexpressed *oxyR*, encoding the master regulator of the oxidative stress response [27], in *C. glutamicum* Δ*hdpA*Δ*ldh*(pEKEx3-*mgsA*-*yqhD*-*gldA*) and analyzed the impact on growth and 1,2-PDO production. Overexpression of *oxyR*, increasing the sensitivity to oxidative stress [27], led to a strongly decreased Y_P/S_of 0.05 ± 0.01 mM_1,2−PDO_ mM_glucose_^−1^ whereas deletion of *oxyR* did not significantly impact 1,2-PDO production after 72 h (*p* > 0.1 regarding the yield), but inhibited growth and glucose uptake (Figure S3). Since the deletion of *mshA* increased the Y_P/S_ strongly, these results may indicate that in *C. glutamicum* Δ*hdpA*Δ*ldh*(pEKEx3-*mgsA*-*yqhD*-*gldA*) a significant portion of methylglyoxal is rerouted to the central carbon metabolism with MSH as reaction partner.

### The metabolome of *C. glutamicum* comprises lactoylmycothiol

We expected that the detoxification of methylglyoxal with MSH would yield lactoylmycothiol as intermediate and therefore applied high-resolution LC-MS-Qtof metabolomics to analyze cell extracts of *C. glutamicum* WT(pEKEx3), *C. glutamicum* Δ*hdpA*Δ*ldh*(pEKEx3-*mgsA*-*yqhD*-*gldA*), *C. glutamicum* Δ*hdpA*Δ*ldh*Δ*dld*(pEKEx3-*mgsA*-*yqhD*-*gldA*) and *C. glutamicum* Δ*hdpA*Δ*ldh*Δ*mshA*(pEKEx3-*mgsA*-*yqhD*-*gldA*) from the production phase. In the analysis at negative ionization, for extracts of the WT(pEKEx3), *C. glutamicum* Δ*hdpA*Δ*ldh*(pEKEx3-*mgsA*-*yqhD*-*gldA*) and its derivative carrying the *dld* deletion, accumulation of a metabolite at Rt 11.1 min was observed. Major mass peaks at m/z = 593.1429 and m/z = 557.1657 and respective expected ^13^C isotope peaks were detected. These correspond with high accuracy (δ 0.7 ppm and δ 0.2 ppm, respectively) to (C_20_H_34_N_2_O_14_S + ^35^Cl)^−^ and (C_20_H_33_N_2_O_14_S)^−^. C_20_H_34_N_2_O_14_S is the sum formula of lactoylmycothiol. In a ms/ms-analysis the (C_20_H_33_N_2_O_14_S)^−^ ion was fragmented. Based on the accurate masses of major fragment ions, we propose that the observed C_20_H_34_N_2_O_14_S metabolite is lactoylmycothiol (Figure S4). In contrast to extracts of *C. glutamicum* Δ*hdpA*Δ*ldh*(pEKEx3-*mgsA*-*yqhD*-*gldA*), we neither detected MSH, mycothione, and the MSH precursor glucosaminyl-myo-inositol nor lactoylmycothiol in extracts of *C. glutamicum* Δ*hdpA*Δ*ldh*Δ*mshA*(pEKEx3-*mgsA*-*yqhD*-*gldA*) (Figure S5).

## Discussion

Genome-reduced strains have been engineered to improve properties of microbes for industrial application, to study the physiological role of gene sets and to indentify essential genes to eventually design minimal genomes with streamlined functions [28, 36]. This concept has also been applied to the industrially relevant bacterium *C. glutamicum* which yielded a strain genealogy varying in the degree of genome reduction [30]. This genome-reduced strain library was recently utilized to screen for strains with improved heterologous secretion of cutinase [31]. In this study, we harnessed the genome-reduced derivative *C. glutamicum* PC2 [30] and its parental strain GRS [29] to unravel metabolic bottlenecks for the production of the industrial relevant bulk chemical 1,2-PDO.

The reconstruction of the previously described genetic modifications [22] to enable 1,2-PDO production in *C. glutamicum* PC2 led to reduced product formation accompanied by the accumulation of lactate. In *C. glutamicum* three enzymes are known, which are responsible for synthesis and utilization of lactate. The NADH-dependent l-lactate dehydrogenase LdhA, encoded by *ldh*, which is mainly responsible for l-lactate formation under excess of NADH [22, 37]. Further, *C. glutamicum* harbors quinone-dependent l- and d-lactate dehydrogenases (LldD and Dld), which are specific for the respective enantiomer and essential for its utilization [32, 38]. *C. glutamicum* PC2Δ*hdpA*Δ*ldh*(pEKEx3-*mgsA*-*yqhD*-*gldA*) lacks LdhA and due to the genome reduction Dld, as well. The applied enzyme assay identified accumulating lactate to be the d-enantiomer. These results suggested the presence of a metabolic route relevant for 1,2-PDO synthesis and with d-lactate as intermediate which was interrupted by the inactive Dld in the PC2 background. Further, no other genes encoding proteins obviously related to the described production pathway could be identified in the PC2 background (Table S2). However, the PC2-derivative showed higher accumulation of lactate and less propanediol compared to the wildtype background harboring the same genetic modification in addition to *dld* deletion (50 ± 1 mM vs. 10 ± 2 mM lactate and 24 ± 1 mM vs. 20 ± 7 mM propanediol; Figs. 2B and 3C and D).

Methylglyoxal is a precursor for 1,2-PDO synthesis but it also represents a cytotoxic intermediate leading to protein inactivation and oxidative stress [23, 24]. In *E. coli* and other organisms methylglyoxal is detoxified with GSH, forming the intermediate S-lactoylglutathione, which is further metabolized via the glyoxalase system GloAB to d-lactate [23, 24, 39]. In *actinobacteria*, such as *C. glutamicum*, the low molecular weight thiol MSH is present instead of GSH [25], however, a glyoxalase-like system based on MSH has not been identified so far. Recently, in the actinobacterium *Streptomyces coelicolor* a Ni^2+^-activated and MSH-dependent glyoxalase I enzyme was described [40]. However, BLASTx analysis [41] did not identify a homolog in *C. glutamicum* (data not shown). Also the deletion of the annotated *gloA* and *gloB* homologs cg1073 as well as cg0071, cg1482 and cg1856 in *C. glutamicum* Δ*hdpA*Δ*ldh*(pEKEx3-*mgsA*-*yqhD*-*gldA*) did not influence growth, glucose consumption and product formation. Therefore, the identification of the glyoxylase system remains elusive and has to be elucidated in future studies. It was shown that overexpression of *mshA*, encoding a glycosyltransferase catalyzing the first step of MSH synthesis, and deletion of the gene *mstX*, encoding a MSH transferase, affected the robustness of *C. glutamicum* towards methylglyoxal [33, 42]. Taken together, these results indicate that, in analogy to GSH-dependent systems, methylglyoxal in *C. glutamicum* is detoxified with MSH via S-lactoylmycothiol by an unknown glyoxalase-like system yielding d-lactate. Notably, LC-MS-Qtof-based analysis identified major mass peaks characteristic for lactoylmycothiol, MSH and glucosaminyl-myo-inositol in cell extracts of the wildtype, *C. glutamicum* Δ*hdpA*Δ*ldh*(pEKEx3-*mgsA*-*yqhD*-*gldA*) and its derivative with inactivated d-lactate dehydrogenase. For lactoylmycothiol, the exact mass and isotope pattern matches the expected sum formula C_20_H_34_N_2_O_14_S, and, although alternative structures cannot be excluded, the observed fragmentation pattern is in accordance with the proposed lactoylmycothiol structure. Moreover, in *C. glutamicum* Δ*hdpA*Δ*ldh*Δ*mshA*(pEKEx3-*mgsA*-*yqhD*-*gldA*), with inactive MSH synthase, lactoylmycothiol and both precursors were not detectable. These findings support the presence of the MSH-dependent detoxification system converting methylglyoxal with MSH to d-lactate in *C. glutamicum*.

The secretion of up to 24 ± 1 mM of d-lactate by *C. glutamicum* Δ*hdpA*Δ*ldh*Δ*dld*(pEKEx3-*mgsA*-*yqhD*-*gldA*) indicated an increased rerouting of methylglyoxal back into the central carbon metabolism in the parental strain with active Dld. Consequently, to improve the precursor availability, we inactivated MshA which significantly improved 1,2-PDO production. *C. glutamicum* Δ*hdpA*Δ*ldh*Δ*mshA*(pEKEx3-*mgsA*-*yqhD*-*gldA*) showed a Y_P/S_ of 0.53 ± 0.01 mM_1,2−PDO_ mM_glucose_^−1^ which is 56% higher compared to *C. glutamicum* Δ*hdpA*Δ*ldh*(pEKEx3-*mgsA*-*yqhD*-*gldA*) and represents the highest reported value for *C. glutamicum*. Also in *E. coli* inactivation of the methylglyoxal detoxification system by deletion of *gloA* proved to be beneficial for 1,2-PDO production from glucose [43].

It should be noted, that the strain lacking *mshA* showed reduced growth and glucose consumption (Figure S2) which might indicate an impaired stress tolerance. In the closely related *Mycobacterium tuberculosis* MSH is essential for survival whereas for *M. smegmatis* it is not [44]. For *E. coli* GSH is dispensable, however, GSH-deficiency results in an increased sensitivity towards oxidative stress [45]. Also in *C. glutamicum* MSH synthesis is not essential but its absence was shown to increase the sensitivity against oxidative stress and some toxic compounds indicated by reduced growth and cell viability [26, 42, 46]. Notably, a MSH-deficient *C. glutamicum* strain, lacking *mshC*, possessed impaired growth in bioreactor cultivations at a pO_2_ of 30%, whereas at a pO_2_ level of 20% this growth defect was abolished [46]. The relevance of a proper functioning oxidative stress response for 1,2-PDO production is also indicated by the result that overexpression of *oxyR* in *C. glutamicum* Δ*hdpA*Δ*ldh*(pEKEx3-*mgsA*-*yqhD*-*gldA*) strongly diminished 1,2-PDO production. OxyR is the master regulator of the oxidative stress response in *C. glutamicum* [27] and under unstressed conditions this transcriptional regulator is acting as a repressor of its target genes (e.g. *katA* encoding catalase). Deletion of *oxyR* resulted in an increased resistance of the *C. glutamicum* mutant to hydrogen peroxide whereas its overexpression completely inhibited growth in the presence of H_2_O_2_ [47]. Therefore, a carefully adjusted oxygen transfer into the culture broth might be crucial for scale-up of fermentation processes based on MSH-deficient 1,2-PDO production strains.

## Conclusion

Libraries with genome-reduced strains provide a valuable tool to identify novel targets for metabolic engineering. In this study, the genetic background of the genome-reduced *C. glutamicum* PC2 strain was the basis to disclose the pathway for detoxification of methylglyoxal with MSH to d-lactate and its relevance for 1,2-PDO production. Subsequent inactivation of this competing pathway significantly improved 1,2-PDO production in *C. glutamicum*. Regarding the detoxification of methylglyoxal, the low molecular weight thiol MSH in *C. glutamicum*, seems to play a role comparable to GSH in other organism, such as *E. coli*. However, the enzymatic machinery catalyzing the detoxification process with MSH has to be identified in future studies.

## Materials and methods

### Microorganisms, media and cultivation conditions

All *C. glutamicum* strains and plasmids used in this work are listed in Table 1. *Escherichia coli* DH5α [48] was used as host for plasmid construction and was grown in lysogeny broth (LB) or in 2x TY complex medium [49] at 37 °C and 120 rpm. For precultivation of *C. glutamicum*, LB medium with 2% (w/v) glucose was applied and the main cultivations were carried out in modified CGXII medium [22]. *C. glutamicum* was cultivated aerobically in 500 mL baffled shaking flasks with 50 mL medium at 30 °C on a rotary shaker at 120 rpm. For induction of gene expression, 1 mM isopropyl-β-D-thiogalactopyranosid (IPTG) and, if appropriate, kanamycin (25 or 50 µg mL^− 1^) and spectinomycin (100 µg mL^− 1^) were supplemented to the medium. For solid medium plates 18 g agar-agar L^− 1^ were added to the liquid medium.

**Table 1:** Strains and plasmids used in this study

Strain or plasmid	Relevant characteristics	Source or reference
C. glutamicum strains		
GRS	Prophage-cured ATCC13032 via in-frame deletion of prophages CGP1 (cg1507-cg1524), CGP2 (cg1746-cg1752) and CGP3 (cg1890-cg2071) with additional in-frame deletions of the insertion elements ISCg1 and ISCg2	[29]
PC2	GRS with in-frame deletions of cg0414-0440, cg0635-0646, cg0704-0748, cg0822-0845, cg1018-1033, cg1172-1213, cg1219-1305 and cg1340-1352	[30]
Δ*hdp*AΔ*ldh*	*C. glutamicum* WT (ATCC13032) with in-frame deletions of *hdpA* (cg2474) and *ldh* (cg3219)	[22]
GRSΔ*hdpA*Δ*ldh*	GRS with in-frame deletions of *hdpA* (cg2474) and *ldh* (cg3219)	This study
PC2Δ*hdpA*Δ*ldh*	PC2 with in-frame deletions of *hdpA* (cg2474) and *ldh* (cg3219)	This study
Δ*hdpA*Δ*ldh*Δ*dld*	*C. glutamicum* WTΔ*hdp*AΔ*ldh* with in-frame deletions of *dld* (cg1027)	This study
Δ*hdpA*Δ*ldh*Δ*mshA*	*C. glutamicum* WTΔ*hdpA*Δ*ldh* with in-frame deletions of *mshA* (cg0481)	This study
Δ*hdpA*Δ*ldh*Δ*oxyR*	*C. glutamicum* WTΔ*hdpA*Δ*ldh* with in-frame deletions of *oxyR* (cg2109)	This study
Δ*hdpA*Δ*ldh*Δcg0071	*C. glutamicum* WTΔ*hdpA*Δ*ldh* with in-frame deletions of cg0071 (proposed *gloB* homolog)	This study
Δ*hdpA*Δ*ldh*Δcg1073	*C. glutamicum* WTΔ*hdpA*Δ*ldh* with in-frame deletions of cg1073 (proposed *gloA* homolog)	This study
Δ*hdpA*Δ*ldh*Δcg1426	*C. glutamicum* WTΔ*hdpA*Δ*ldh* with in-frame deletions of cg1426 (coding for Xi-class mycothiol S-transferase)	This study
Δ*hdpA*Δ*ldh*Δcg1482	*C. glutamicum* WTΔ*hdpA*Δ*ldh* with in-frame deletions of cg1482 (proposed *gloB* homolog)	This study
Δ*hdpA*Δ*ldh*Δcg1856	*C. glutamicum* WTΔ*hdpA*Δ*ldh* with in-frame deletions of cg1856 (proposed *gloB* homolog)	This study
**Plasmids**		
pK19*mobsacB*	Mobilizable *E. coli* vector for the construction of deletion and insertion mutants, Kan^R^, *sacB*, *lacZα* with multiple cloning site, *oriV*, *oriT*	[50]
pK19*mobsacB*-Δ*hdpA*	Kan^R^, pK19*mobsacB* with the deletion construct for *hdpA* (cg2474)	[22]
pK19*mobsacB*-Δ*ldh*	Kan^R^, pK19*mobsacB* with the deletion construct for *ldh* (cg3219)	[51]
pK19*mobsacB*-Δ*dld*	Kan^R^, pK19*mobsacB* with the deletion construct for *dld* (cg1027)	This study
pK19*mobsacB*-Δ*mshA*	Kan^R^, pK19*mobsacB* with the deletion construct for *mshA* (cg0481)	This study
pK19*mobsacB*-Δ*oxyR*	Kan^R^, pK19*mobsacB* with the deletion construct for *oxyR* (cg2109)	This study
pK19*mobsacB*-Δcg0071	Kan^R^, pK19*mobsacB* with the deletion construct for cg0071 (proposed *gloB* homolog)	This study
pK19*mobsacB*-Δcg1073	Kan^R^, pK19*mobsacB* with the deletion construct for cg1073 (proposed *gloA* homolog)	This study
pK19*mobsacB*-Δcg1426	Kan^R^, pK19*mobsacB* with the deletion construct for cg1426 (coding for Xi-class mycothiol S-transferase)	This study
pK19*mobsacB*-Δcg1482	Kan^R^, pK19*mobsacB* with the deletion construct for cg1482 (proposed *gloB* homolog)	This study
pK19*mobsacB*-Δcg1856	Kan^R^, pK19*mobsacB* with the deletion construct for cg1856 (proposed *gloB* homolog)	This study
pEKEx3-*mgsA*-*yqhD*-*gldA*	Spec^R^; derived from pEKEx3 for IPTG-inducible overexpression of *mgsA*, *yqhD* and *gldA* from *E. coli* with artificial ribosome binding site in front of each gene	[22]
pVWEx1	Kan^R^; *C. glutamicum*/*E. coli* shuttle vector for regulated gene expression (P_tac_, *lacI*^q^, pHM1519, oriV_C.g_., oriV_E.c_.)	[52]
pVWEx1-*oxyR*	Kan^R^; derived from pVWEx1 for IPTG-inducible overexpression of *oxyR* (cg2109) from *C. glutamicum* with artificial ribosome binding site in front of the gene	This study

^R^resistance gene; ^q^Quantity

### Recombinant DNA work and construction of *C. glutamicum* deletion mutants

Oligonucleotides (Table S1) were purchased either from Eurofins MWG Operon (Ebersberg, Germany), metabion international AG (Planegg, Germany) or Sigma-Aldrich Chemie GmbH (Steinheim, Germany). The construction of plasmids (Table 1) was carried out as described in [9]. Purification and isolation of PCR products, plasmids and genomic DNA was conducted via “NucleoSpin® Gel and PCR Clean-up”, “NucleoSpin® Plasmid” and “NucleoSpin® Microbial DNA”, respectively, as recommended by MACHEREY-NAGEL GmbH & Co. KG (Düren, Germany). Enzymes were all purchased from New England Biolabs GmbH [(NEB), Frankfurt am Main, Germany] and used as recommended by the manufacturer. For cloning of pVWEx1-*oxyR*, pVWEx1 [52] was linearized via BamHI and the *oxyR* gene was PCR-amplified with primers #16 and #17 from genomic *C. glutamicum* DNA by Phusion® High-Fidelity DNA Polymerase. pVWEx1 and the insert were fused via Gibson assembly [53]. To construct pK19*mobsacB* [50] derivatives the plasmid was linearized via SmaI and the two homologous flanks for recombination were amplified via PCR, respectively. E.g. for the deletion of the *mshA* gene the primer pairs #19 + 20 and #21 + 22 (Table S1) were used. Eventually, the flanks and linearized pK19*mobsacB* were fused via Gibson assembly [53]. For subsequent transformation of *E. coli* DH5α the calcium chloride method was conducted [49]. Positive clones were identified by colony PCR applying Quick-Load R® Taq 2X Master Mix, validated via restriction digestion with appropriate enzymes and eventually the correct sequence of the inserts was confirmed via Sanger sequencing by Microsynth Seqlab GmbH (Göttingen, Germany).

For transformation and markerless gene deletions in *C. glutamicum* GRS and PC2 derivatives the procedure of [22] was applied: Electrocompetent cells [54] were transformed via electroporation [6] and, in case of gene deletions via pK19*mobsacB*, a subsequent two-step homologous recombination procedure followed [6]. All WTΔ*hdpA*Δ*ldh* derivatives were engineered as described in [9]: Electrocompetent cells [55] were transformed via electroporation [56] and the subsequent two-step homologous recombination was carried out as described previously [50]. Successful gene deletions were confirmed via colony PCR as described above with the respective primer pairs listed in Table S1.

### Analytical methods

#### Determination of biomass

OD_600_ (optical density at 600 nm) was measured with a spectrophotometer (either V-1200 of VWR International, Darmstadt, Germany or ULTROSPEC® 10 of Biochrom, Holliston, MA, United States) by diluting the samples into an OD_600_ range of 0.05 to 0.3. Cell dry weight (CDW) was calculated via a correlation factor of either 0.25 g L^− 1^ (V-1200) or 0.23 g L^− 1^ (ULTROSPEC® 10) per OD_600_ of 1. The growth rates (h^− 1^) were determined by linear regression in a semi-logarithmic blot and maximizing the coefficient of determination (R^2^) in the exponential growth phase. At the given time points 1 mL samples were taken, centrifuged (10 min, 16,000 or 21,300 x g) and the resulting supernatants were stored at -20 °C for further analysis.

#### GC-MS and HPLC analysis

The GC-MS-based quantification of 1,2-PDO and the HPLC measurement of glucose and lactate shown in Fig. 1 was conducted as described before [22].

Elsewhere, glucose, 1,2-PDO, lactate and acetol were determined via a modified HPLC method as described in [9], employing a 1260 Infinity II system (Agilent Technologies, Waldbronn, Germany), equipped with a Hi-Plex H column (7.7 × 300 mm, 8 μm) and a Hi-Plex H guard cartridge (3 × 5 mm, 8 μm) kept at 30 °C. As mobile phase 5 mM sulfuric acid (H_2_SO_4_) in water was applied with a flow rate of 0.4 mL min^− 1^, while signal acquisition was conducted via refractive index detector (RID) hold at 30 °C.

#### Metabolomics via LC-MS

For LC-MS analyses cell extracts were prepared as previously described for *C. glutamicum* [57]. From cultures grown as described above for HPLC analysis, cells were harvested via centrifugation (5 min, 3,000 x g, 4 °C), washed once with 0.5 volumes of saline (9 g L^− 1^ sodium chloride in water) and again centrifuged. The cell pellet was resuspended with boiling water to adjust a concentration of approximately 125 g CDW L^− 1^. This suspension was incubated three times in a bath with boiling water for 2 min with intermittent vortexing of 5 s. The cell extracts were collected by centrifugation (10 min, 20,000 x g, 4 °C), aliquoted and stored at -80 °C for further analysis.

LC-MS analyses were performed on a bio-inert 1290 series UHPLC system which was interfaced to a Q-TOF mass spectrometer (G6546AA, Agilent Technologies) via a dual Agilent jet stream electrospray ion source. MassHunter LC/MS Data Acquisition (version 10.1) and Qualitative Analysis software (version 10.0) from Agilent Technologies was used for data acquisition and data evaluation, respectively. The mass spectrometer was operated in low mass range (*m/z* 1700), Gas temp 325 °C, VCap 4000 V, Nozzle Voltage 2000 V. The instrument was auto tuned and calibrated according to manufacturer´s recommendations using ESI-L tuning mix (Agilent Technologies).

Extracts (5 µL injection volume) were separated on a InfinityLab Poroshell 120 HILIC-Z (150 × 2.1 mm, 2.7 μm particle size) using acetonitrile /10 mM CH_3_COONH_4_ in milli-Q H_2_O (pH 9.2), 9:1, v/v and acetonitrile /10 mM CH_3_COONH_4_ in milli-Q H_2_O (pH 9.2), 1:9, v/v, as eluents A and B, respectively. The following binary gradient program at a flow rate of 200 µL min^− 1^ was applied: 0–1 min isocratic 0% B, 1–31 min linear from 0 to 75% B, 31–35 min linear from 75 to 100% B, 35–40 min isocratic 100% B; 40–50 min linear from 100 to 0% B, 50–65 min, isocratic, 0% B. The column temperature was maintained at 40 °C and the autosampler temperature at 4 °C. Eluting compounds were detected from *m/z* 50-1700 in negative ion mode. Mass spectra were acquired in centroid mode using an acquisition rate of 1.4 spectra per second.

Collision-induced dissociation (CID) mass spectra were acquired in targeted MS/MS mode. Precursor ions were isolated by the quadrupole using an isolation width of *m/z* 4 and fragmented inside the collision cell by applying collision energies in the range of 10, 20, and 30 V and nitrogen as collision gas. Fragment ions were detected from *m/z* 100–1000. For reference mass correction a solution of purine (5 µM) and hexakis-(2,2,3,3-tetrafluoropropoxy)phosphazine (2.5 µM) in acetonitrile/water, 95/5 (v/v) % was continuously introduced through the second sprayer of the dual ion source at a flow rate of 10 µL min^-1^ using an isocratic HPLC pump (G7110B, Agilent Technologies) equipped with a 1:100 splitting.

Metabolites were annotated according to high resolution mass: Lactoylmycothiol, C_20_H_34_N_2_O_14_S, Rt = 11.1 min, (m-H)^−^: 557.1657 (expect: 557.1658, δ 0.2 ppm), m-^35^Cl^−^: 593.1429 (expect: 593.1425, δ 0.7 ppm), (m-C_3_H_6_O_3_-H)^−^: 467.1338 (co-eluting, putative in-source fragment, expect: 467.1341), MSH C_17_H_30_N_2_O_12_S, Rt = 11.4 min, (m-H)^−^: 485.1450 (expect: 485.1447, δ 0.7 ppm), mycothione, Rt = 16.1 min C_34_H_57_N_4_O_24_S_2_, (m-H)^−^: 969.2807 (expect: 969.2809, δ 0.2 ppm), glucosaminyl-myo-inositol C_12_H_22_NO_10_ Rt = 14.7 min (m-H)^−^: 400.1459 (expect: 400.1460, δ 0.3 ppm).

#### Enzyme assay specific for d-lactate

For the specific, enzymatic determination of d-lactate the “D-LACTIC ACID (D-LACTATE) (*Rapid*) Assay Kit (K-DATE)” from Megazyme Ltd. (Wicklow, Ireland) was purchased and applied as recommended by the manufacturer. The assay was conducted in a 96-well format with a Tecan Spark and SparkControl V 2.3 (Tecan Group Ltd., Männedorf, Switzerland), while for quantification an external calibration curve ranging from 0 to 0.15 g L^− 1^ was prepared with a d-lactic acid standard solution.

#### Determination of significance

The significance (p-value) was calculated via “two-sample t-test assuming equal variances” in Excel 2016 (Microsoft Corporation, Redmond, Washington, USA).

### Electronic supplementary material

Below is the link to the electronic supplementary material.

Supplementary Material 1

Supplementary Material 2

## Data Availability

No datasets were generated or analysed during the current study.
