# Supplementary material for: A genome-reduced Corynebacterium glutamicum derivative discloses a hidden pathway relevant for 1,2-propanediol production
Source: Microb Cell Fact. 2024 Feb 24;23:62. doi: 10.1186/s12934-024-02337-w (PMC10893638; doi:10.1186/s12934-024-02337-w)
Supplement: Supplementary file 2 — Supplementary Material 2 [file 12934_2024_2337_MOESM2_ESM.docx]

**Supplementary data**

**A genome reduced *Corynebacterium glutamicum* derivative discloses a hidden pathway relevant for 1,2-propanediol production**

**Daniel Siebert, Erich Glawischnig, Marie-Theres Wirth, Mieke Vannahme, Álvaro Salazar-Quirós, Annette Weiske, Ezgi Saydam, Dominik Möggenried, Volker F. Wendisch, Bastian Blombach**

# Content

1. Comparison of HPLC-determined lactate and d-lactate specific determination via enzymatic assay in supernatants of *C. glutamicum* Δ*hdpA*Δ*ldh*Δ*dld*(pEKEx3-*mgsA*-*yqhD*-*gldA*).
2. Cultivation parameters of *C. glutamicum* Δ*hdpA*Δ*ldh*(pEKEx3-*mgsA*-*yqhD*-*gldA*) and its derivatives harboring the single gene deletion of either *mshA*, cg1426, cg1073, cg0071, cg1482 or cg1856.
3. Comparison of *C. glutamicum* Δ*hdpA*Δ*ldh*(pEKEx3-*mgsA*-*yqhD*-*gldA*) with additional deletion or overexpression of *oxyR.*
4. Proposed structures for fragment ions of lactoylmycothiol.
5. Extracted ion chromatograms for compounds accumulating in *C. glutamicum* Δ*hdpA*Δ*ldh*Δ*dld*(pEKEx3-*mgsA*-*yqhD*-*gldA*) and in *C. glutamicum* Δ*hdpA*Δ*ldh*Δ*mshA*(pEKEx3-*mgsA*-*yqhD*-*gldA*).
6. Oligonucleotides used in this study.


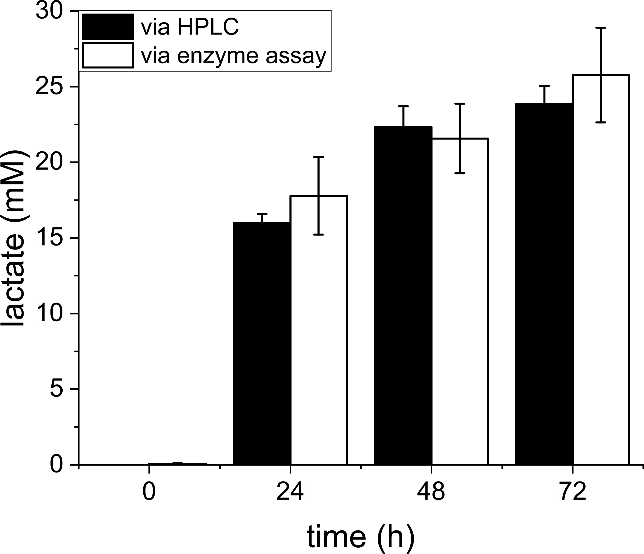


Figure S1: Lactate determined via HPLC (black) and via D-lactate specific enzyme assay (white) in the supernatants of the strain *C. glutamicum* Δ*hdpA*Δ*ldh*Δ*dld*(pEKEx3-*mgsA*-*yqhD*-*gldA*), cultivated in shaking flasks with modified CGXII minimal medium and glucose as sole carbon source. Error bars represent the standard deviation of the mean values of three biological replicates.


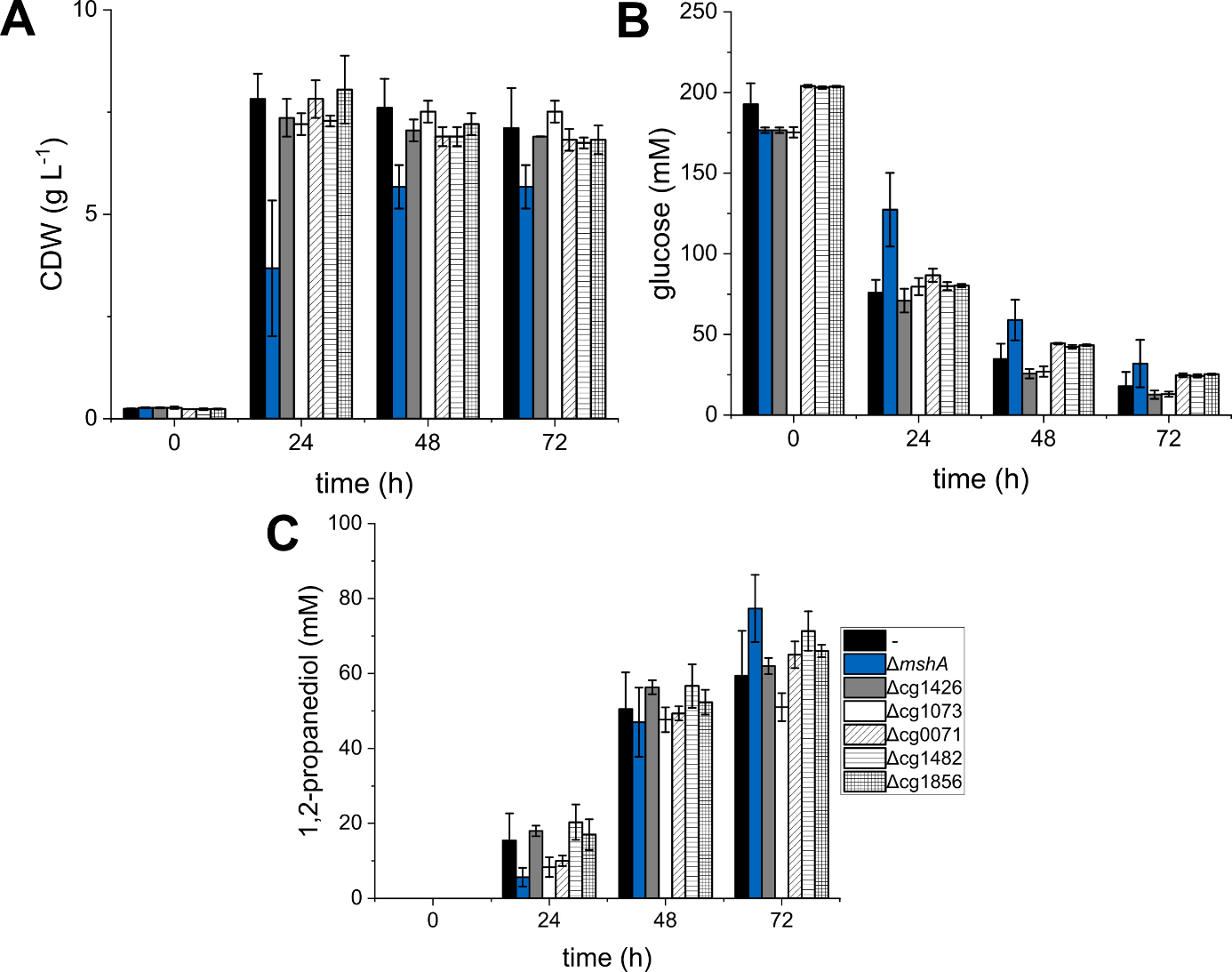


Figure S2: (A) Growth, (B) glucose consumption and (C) 1,2-propandiol accumulation of *C. glutamicum* Δ*hdpA*Δ*ldh*(pEKEx3-*mgsA*-*yqhD*-*gldA*) (-), *C. glutamicum* Δ*hdpA*Δ*ldh*Δ*mshA*(pEKEx3-*mgsA*-*yqhD*-*gldA*) (Δ*mshA*), *C. glutamicum* Δ*hdpA*Δ*ldh*Δcg1426(pEKEx3-*mgsA*-*yqhD*-*gldA*) (Δcg1426), *C. glutamicum* Δ*hdpA*Δ*ldh*Δcg1073(pEKEx3-*mgsA*-*yqhD*-*gldA*) (Δcg1073), *C. glutamicum* Δ*hdpA*Δ*ldh*Δcg0071(pEKEx3-*mgsA*-*yqhD*-*gldA*) (Δcg0071), *C. glutamicum* Δ*hdpA*Δ*ldh*Δcg1482(pEKEx3-*mgsA*-*yqhD*-*gldA*) (Δcg1482) and *C. glutamicum* Δ*hdpA*Δ*ldh*Δcg1856(pEKEx3-*mgsA*-*yqhD*-*gldA*) (Δcg1856) cultivated in shaking flasks with modified CGXII minimal medium and glucose as sole carbon and energy source. Error bars represent the standard deviation of the mean values of three biological replicates.


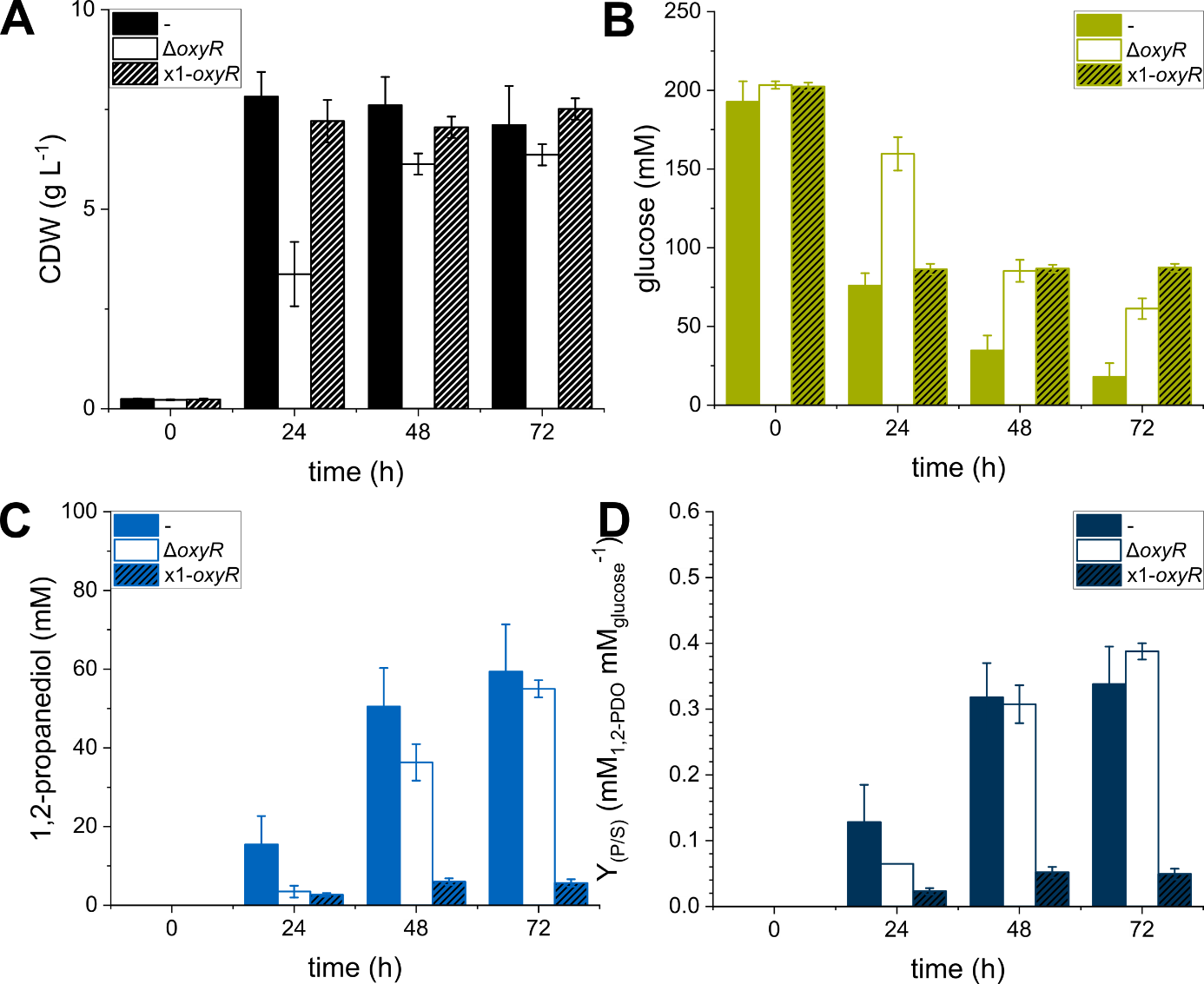


Figure S3: (A) Growth (black), (B) glucose consumption (green), (C) 1,2-propandiol (light blue) accumulation and (D) product yields (dark blue) of *C. glutamicum* Δ*hdpA*Δ*ldh*(pEKEx3-*mgsA*-*yqhD*-*gldA*) (-), *C. glutamicum* Δ*hdpA*Δ*ldh*Δ*oxyR*(pEKEx3-*mgsA*-*yqhD*-*gldA*) (Δ*oxyR*) and *C. glutamicum* Δ*hdpA*Δ*ldh*(pEKEx3-*mgsA*-*yqhD*-*gldA*)(pVWEx1-*oxyR*) (x1-*oxyR*) cultivated in shaking flasks with modified CGXII minimal medium and glucose as sole carbon and energy source. Error bars represent the standard deviation of the mean values of three biological replicates.

**Figure S4:** Putative lactoylmycothiol (**1**): Collision-induced dissociation (CID) mass spectrum, ESI (-), collision energy 20 V, sample *C. glutamicum* Δ*hdpA*Δ*ldh*Δ*dld*(pEKEx3-*mgsA*-*yqhD*-*gldA*) after 48 h, 12.244 min, parent ion (red diamond) m/z 557.16, C_20_H_33_N_2_O_14_S^-^, m/z 100 – 585 shown; (**2)** to (**6)**: proposed structures for fragment ions.

**Figure S5:** Extracted ion chromatograms (negative ionization) representative for compounds accumulating in *C. glutamicum* Δ*hdpA*Δ*ldh*Δ*dld*(pEKEx3-*mgsA*-*yqhD*-*gldA*) (left, ion trace in blue; A1-D1) and not detected in *C. glutamicum* Δ*hdpA*Δ*ldh*Δ*mshA*(pEKEx3-*mgsA*-*yqhD*-*gldA*) (right, ion trace in red; A2-D2) at 48 h. (A) m/z 557.16 – 557.17, representative for the monoisotopic mass of lactoylmycothiol (M-H)^-^, C_20_H_33_N_2_O_14_S^-^, (B) m/z 485.14 – 485.15, representative for the monoisotopic mass of mycothiol (M-H)^-^, C_17_H_29_N_2_O_12_S^-^, (C) m/z 969.28 – 969.29, representative for the monoisotopic mass of mycothione (M-H)^-^, C_34_H_57_N_4_O_24_S_2_^-^, (D) m/z 340.12 – 340.13, representative for the monoisotopic mass of the mycothiol precursor glucosaminyl-myo-inositol (M-H)^-^, C_12_H_22_NO_10_^-^.

## Oligonucleotides used in this study

Table S1: Oligonucleotides used in this study

| **#** | **Oligonucleotide name** | **Sequence (5’ 🡪 3’)** | **Purpose** |
| --- | --- | --- | --- |
| 1 | DnagD_fw | GATGAACACGACCGTTGCC | Verification of *hdpA* deletion by PCR |
| 2 | DnagD_rv | GGGTGGTCTTTGAGGAGTTCTTC | Verification of *hdpA* deletion by PCR |
| 3 | ldhfow | TGATGGCACCAGTTGCGATGT | Verification of *ldh* deletion by PCR |
| 4 | ldhrev | CCATGATGCAGGATGGAGTA | Verification of *ldh* deletion by PCR |
| 5 | M13_fw | CGCCAGGGTTTTCCCAGTCACGAC | Verification of pK19*mobsacB*  derivatives by PCR/sequencing |
| 6 | M13_rv | AGCGGATAACAATTTCACACAGGA | Verification of pK19*mobsacB*  derivatives by PCR/sequencing |
| 7 | pK19lacZ_fw | ATGACCATGATTACGCCAAGCTTG | Verification of pK19*mobsacB*  derivatives by PCR/sequencing |
| 8 | pK19lacZ_rv | TTAGCAGCCCTTGCGCC | Verification of pK19*mobsacB*  derivatives by PCR/sequencing |
| 9 | pK19lacI_fw | TCAGTGAGCGAGGAAGCG | Verification of pK19*mobsacB*  derivatives by PCR/sequencing |
| 10 | pVWEx1_fw | ATAACAATTTCACACAGGAAACAGAATTAAAAGATATG | Verification of pVWEx1  derivatives by PCR/sequencing |
| 11 | pVWEx1_rv | TTAATCTGTATCAGGCTGAAAATCTTCTCTCA | Verification of correct pVWEx1  derivatives by PCR/sequencing |
| 12 | pX2_Seq_fw | CACTCCCGTTCTGGATAATG | Confirmation of pEKEx3-*mgsA*-*yqhD*-*gldA* in *C. glutamicum* by PCR |
| 13 | pX2_Seq_rv | GCTACGGCGTTTCACTTCTG | Confirmation of pEKEx3-*mgsA*-*yqhD*-*gldA* in *C. glutamicum* by PCR |
| 14 | mgsA_Seq_fw | ATAATCCAGTCGCCGCATTT | Confirmation of pEKEx3-*mgsA*-*yqhD*-*gldA* in *C. glutamicum* by PCR |
| 15 | gldA_Seq_rv | ATCTCATTTTGCGAACATTC | Confirmation of pEKEx3-*mgsA*-*yqhD*-*gldA* in *C. glutamicum* by PCR |
| 16 | oxyR_fw_x1 | CCTGCAGGTCGACTCTAGAGGAAAGGAGGCCCTTCAGAT-GAGCAATAAAGAGTACCGGCC | Construction of pVWEx1-*oxyR* |
| 17 | oxyR_rv_x1 | GAGCTCGGTACCCGGGGATCTTACTGCGCTACCGCGAC | Construction of pVWEx1-*oxyR* |
| 18 | oxyR_Seq | GGCATGAAGGAAATCCCCCT | Verification of pVWEx1-*oxyR* by sequencing |
| 19 | mshA_upstrm_fw_pK19 | GTCGACTCTAGAGGATCCCCAGGTTGGGGTGTGCTTTTTCATTAC | Construction of pK19*mobsacB*-Δ*mshA* |
| 20 | mshA_upstrm_rv | GGGTAGGTGATTTGAATTTGTAGCTACGCGCATGCCC | Construction of pK19*mobsacB*-Δ*mshA* |
| 21 | mshA_dwnstrm_fw | ACAAATTCAAATCACCTACCCAACGAAAATGTCGACGGTGAAAC | Construction of pK19*mobsacB*-Δ*mshA* |
| 22 | mshA_dwnstrm_rv_pK19 | TGAATTCGAGCTCGGTACCCGCCGGTGCGATCGACAA | Construction of pK19*mobsacB*-Δ*mshA* |
| 23 | DmshA_fw | CGTAGCTGTTCGACGGTGG | Verification of *mshA* deletion by PCR |
| 24 | DmshA_rv | TTGAGGCATTCGGTGCGT | Verification of *mshA* deletion by PCR |
| 25 | cg1073_upstrm_fw_pK19 | GTCGACTCTAGAGGATCCCCACAAGCAAAAACAGGCCCC | Construction of pK19mobsacB-Δcg1073 |
| 26 | cg1073_upstrm_rv | GGGTAGGTGATTTGAATTTGTGTGCTGAAGTCGTGCCATAGG | Construction of pK19mobsacB-Δcg1073 |
| 27 | cg1073_dwnstrm_fw | ACAAATTCAAATCACCTACCCCCAGAGGCACTCGCTCAGAT | Construction of pK19mobsacB-Δcg1073 |
| 28 | cg1073_dwnstrm_rv_pK19 | TGAATTCGAGCTCGGTACCCCCCGCTCCACCGAGGTAG | Construction of pK19mobsacB-Δcg1073 |
| 29 | Dcg1073_fw | GGTGAGCTGCTTCTGGCC | Verification of cg1073 deletion by PCR |
| 30 | Dcg1073_rv | ACCCCCAGAAAAGAAGCCTG | Verification of cg1073 deletion by PCR |
| 31 | cg1426_upstrm_fw_pK19 | GTCGACTCTAGAGGATCCCCTCCGATGGACAGTAAAAGACTGG | Construction of pK19mobsacB-Δcg1426 |
| 32 | cg1426_upstrm_rv | GGGTAGGTGATTTGAATTTGTGAACCTTTTCAGAAGTAACTAAGGCCG | Construction of pK19mobsacB-Δcg1426 |
| 33 | cg1426_dwnstrm_fw | ACAAATTCAAATCACCTACCCTGGGGCGCCTGCC | Construction of pK19mobsacB-Δcg1426 |
| 34 | cg1426_dwnstrm_rv_pK19 | TGAATTCGAGCTCGGTACCCCGAGTTATCAGTCATGTGCCCC | Construction of pK19mobsacB-Δcg1426 |
| 35 | Dcg1426_fw | AAGCGCACACATTATCCTTGCT | Verification of cg1426 deletion by PCR |
| 36 | Dcg1426_rv | GTCGCGTTGCAGGATGTC | Verification of cg1426 deletion by PCR |
| 37 | dld_upstrm_fw_pK19 | GTCGACTCTAGAGGATCCCCGGGTGTGTTGGGATAACGGAAG | Construction of pK19mobsacB-Δ*dld* |
| 38 | dld_upstrm_rv | GGGTAGGTGATTTGAATTTGTCACAAGGACTGGGCCTAAGTC | Construction of pK19mobsacB-Δ*dld* |
| 39 | dld_dwnstrm_fw | ACAAATTCAAATCACCTACCCGTGCGAAGTCGTGGTGGTC | Construction of pK19mobsacB-Δ*dld* |
| 40 | dld_dwnstrm_rv_pK19 | TGAATTCGAGCTCGGTACCCGAGCGACGGTTGAAGATCTGTC | Construction of pK19mobsacB-Δ*dld* |
| 41 | Ddld_fw | GGAGCACCTGCGCGG | Verification of *dld* deletion by PCR |
| 42 | Ddld_rv | TACCCGGGCAGCGC | Verification of *dld* deletion by PCR |
| 43 | cg0071_upstrm_fw_pK19 | GTCGACTCTAGAGGATCCCCGCGTCGAGGTAGTCCTGTG | Construction of pK19mobsacB-Δcg0071 |
| 44 | cg0071_upstrm_rv | GGGTAGGTGATTTGAATTTGTGATGCGTTCAATAAGCACTGAGCT | Construction of pK19mobsacB-Δcg0071 |
| 45 | cg0071_dwnstrm_fw | ACAAATTCAAATCACCTACCCTCCTATGACAACTGGGTCCGG | Construction of pK19mobsacB-Δcg0071 |
| 46 | cg0071_dwnstrm_rv_pK19 | TGAATTCGAGCTCGGTACCCACTTTCGCTTCAAAGAGCTAGATCAG | Construction of pK19mobsacB-Δcg0071 |
| 47 | Dcg0071_fw | AGCGAAGACTCACCACTCAAC | Verification of cg0071 deletion by PCR |
| 48 | Dcg0071_rv | GATGATCGCCACGTCGACTG | Verification of cg0071 deletion by PCR |
| 49 | cg1482_upstrm_fw_pK19_2 | GTCGACTCTAGAGGATCCCCTTAAAGGAAAATGCCGGAAAATGAAGC | Construction of pK19mobsacB-Δcg1482 |
| 50 | cg1482_upstrm_rv | GGGTAGGTGATTTGAATTTGTTTGCAGACTGCCGTCTAGTTTTAGA | Construction of pK19mobsacB-Δcg1482 |
| 51 | cg1482_dwnstrm_fw | ACAAATTCAAATCACCTACCCGGCGAGGCCTTCTTCGTAGA | Construction of pK19mobsacB-Δcg1482 |
| 52 | cg1482_dwnstrm_rv_pK19_2 | TGAATTCGAGCTCGGTACCCTTGTGCCAGCCTTGATTTTTGC | Construction of pK19mobsacB-Δcg1482 |
| 53 | Dcg1482_fw | CGATCAGTAGCGAGGTCGG | Verification of cg1482 deletion by PCR |
| 54 | Dcg1482_rv | GAATACATAACCAGAAGGCCAAGCATC | Verification of cg1482 deletion by PCR |
| 55 | cg1856_upstrm_fw_pK19 | GTCGACTCTAGAGGATCCCCTGCGATCCACACCCAGG | Construction of pK19mobsacB-Δcg1856 |
| 56 | cg1856_upstrm_rv | GGGTAGGTGATTTGAATTTGTTTCTTGCTGGCACTGTAGGG | Construction of pK19mobsacB-Δcg1856 |
| 57 | cg1856_dwnstrm_fw | ACAAATTCAAATCACCTACCCTTTATACGGACCAGCCGCGA | Construction of pK19mobsacB-Δcg1856 |
| 58 | cg1856_dwnstrm_rv_pK19 | TGAATTCGAGCTCGGTACCCGGTGTTGAAAGAAGAGGGGCTTAG | Construction of pK19mobsacB-Δcg1856 |
| 59 | Dcg1856_fw | GCATCAAGCGCTGGATCATC | Verification of cg1856 deletion by PCR |
| 60 | Dcg1856_rv | TGGTCAGGTTGACGGTCAC | Verification of cg1856 deletion by PCR |
| 61 | oxyR_upstrm_fw_pK19 | GTCGACTCTAGAGGATCCCCCTCCTTGCGCAGCAAAAAGC | Construction of pK19mobsacB-Δ*oxyR* |
| 62 | oxyR_upstrm_rv | GGGTAGGTGATTTGAATTTGTGTACTCTTTATTGCTCATGCCTATAACTATAACG | Construction of pK19mobsacB-Δ*oxyR* |
| 63 | oxyR_dwnstrm_fw | ACAAATTCAAATCACCTACCCATCACCTTGAAGCAAAATGTCGC | Construction of pK19mobsacB-Δ*oxyR* |
| 64 | oxyR_dwnstrm_rv_pK19 | TGAATTCGAGCTCGGTACCCAGGGCTTTGGCCCGG | Construction of pK19mobsacB-Δ*oxyR* |
| 65 | DoxyR_fw | AGCACTTACCGCCAACCG | Verification of *oxyR* deletion by PCR |
| 66 | DoxyR_rv | GGCACCTTCACCACTTTTCTCAC | Verification of *oxyR* deletion by PCR |
